# Attitudes Among Physician Associates Regarding Naloxone Prescribing for the Treatment of Opioid Overdose

**DOI:** 10.7759/cureus.117750

**Published:** 2026-10-10

**Authors:** Amanda Novak, Kirsten N Brondstater

**Affiliations:** 1 Pain/Addiction, Wayspring, Nashville, USA; 2 Family and Community Medicine, Shenandoah University, Winchester, USA

**Keywords:** harm reduction, naloxone, opioid overdose, provider attitudes, qualitative, stigma

## Abstract

Objective

This study aims to explore and better understand current attitudes and barriers among physician associates (PAs) toward naloxone prescribing to inform efforts that support wider adoption of overdose prevention strategies through naloxone prescribing.

Methods

This is a cross-sectional, descriptive survey study with quantitative and qualitative components. This 33-question survey was distributed to PAs who have graduated from an accredited PA program via email and social media.

Results

Ninety PAs across 34 states and 27 specialties completed the survey. Respondents affirm use of naloxone as a life-saving intervention and support its normalization in the community. Results also identified prescribing barriers and potential opportunities to decrease stigma and increase the prescribing rates of naloxone.

Conclusions

PAs demonstrated broadly favorable attitudes toward naloxone and overdose prevention; however, time constraints, concerns regarding patient perceptions, uncertainty regarding state-specific laws, and gaps in confidence were identified as potential barriers to routine prescribing. The results of this survey suggest that further work in developing real-world insights and educating PAs is likely to result in increased naloxone prescribing.

## Introduction

The opioid crisis remains one of the foremost public health challenges in the United States. Prescription opioids have been implicated in the current opioid epidemic. Of the 80,400 total drug overdose deaths reported in 2024 data, approximately 48,422 were associated with opioid use [1]. The opioid crisis has become one of the leading causes of death among adults in the United States, a trend that has worsened over the past several decades [2]. Naloxone is a rapid‑acting opioid antagonist that reverses respiratory depression related to opioid overdose when administered promptly [3]. Making availability widespread is an important factor in its use as a harm reduction tool. The US Department of Health and Human Services (HHS) has recommended the co-prescribing of naloxone with opioid prescriptions since 2018 [3]. The Centers for Disease Control and Prevention (CDC) published guidelines reinforcing the recommendation that clinicians should prescribe naloxone in 2022, particularly to higher-risk patients [4]. The CDC subsequently expanded its guidance and now recommends offering naloxone to all individuals prescribed opioids, those with opioid use disorder, as well as their families and caregivers [5].

The evidence for the role of naloxone in reducing opioid-related deaths has been well documented. Qeadan and Madden analyzed over 1.1 million national electronic health records, finding that naloxone co‑prescribing correlates with reduced overdose events among pain patients [3]. Much of the existing research on naloxone prescribing has focused on its clinical efficacy and patient or caregiver acceptance, particularly in the context of chronic pain and opioid use disorder [6]. It has been suggested that increasing access to naloxone for non‑medical individuals may be an effective intervention in combating opioid overdose deaths, especially in areas with widespread naloxone programs [7].

Multiple studies support that broader naloxone access does not worsen opioid misuse and can motivate protective behavior despite concerns from some individuals that naloxone promotes risky substance use [7,8]. Healthcare providers play an essential role in supporting naloxone availability. Research demonstrates that provider stigma can negatively influence their naloxone prescribing practices [9,10]. Providers' attitudes are influential in their prescribing, while patients' beliefs may determine acceptance of the drug. Perceptions and acceptance of naloxone use and availability are relevant for both patients and prescribers in order to increase naloxone use and availability. The bulk of existing research examining provider perceptions around naloxone prescribing was conducted almost a decade ago when naloxone co-prescribing was first introduced as recommended clinical practice. It is likely that provider attitudes and barriers to naloxone prescribing have changed for reasons including clinical experience, broader familiarity with national practice guidelines, and provider quality metrics related to opioid management.

Little research has been conducted to date that specifically examines naloxone attitudes, barriers, and prescribing practices among physician associates (PAs). PAs play a substantial role in opioid prescribing and are uniquely positioned to prescribe naloxone in primary care and other settings, yet their specific attitudes and any perceived practice barriers have not been examined. This survey-based study aims to explore current attitudes and barriers among PAs toward naloxone prescribing by gathering current, profession-specific insights. Understanding how perceived stigma influences PA decision-making is essential to inform efforts that support wider adoption of overdose prevention strategies through naloxone prescribing.

## Materials and methods

Study design

An original research study was conducted through an online survey to collect and analyze new data on PAs' attitudes and practices regarding naloxone prescribing. Institutional review board (IRB) exemption for the survey research was obtained prior to data collection.

Participants were invited to complete an anonymous online survey hosted on SurveyMonkey. A non-validated, investigator-developed survey (see Appendices) was used due to the lack of existing validated instruments that would adequately address the study objectives. The survey included questions related to current prescribing practices and attitudes toward naloxone use and distribution. Perceived barriers around naloxone prescribing were also captured. Completion of the survey was estimated to take no more than 10 minutes. Respondents remain anonymous unless they voluntarily provide contact information via an email address for follow-up.

Study participants

Survey recruitment was conducted through email outreach and social media platforms targeting PAs. These recruitment platforms were inclusive of LinkedIn, Facebook, AAPA Huddle, Shenandoah University, and Grand Valley State University. These social media platforms were selected in order to obtain a broad range of perspectives while recognizing that the sample is not population-based and may be subject to self-selection and sampling bias. The survey was open for two weeks.

Study analysis

Responses are analyzed qualitatively to identify trends and emerging themes related to naloxone prescribing behaviors and perceptions. As an exploratory instrument only, the survey is not intended to provide psychometrically validated measures of attitudes or to support causal inference.

## Results

While the number of individuals who received the survey is not known due to the nature of the social media-based recruiting strategy, a total of 90 respondents who met the inclusion criteria completed the survey. The survey was completed with 95.5% (n=86) of those who responded providing answers to all questions. Additional comments were provided by 32.2% of respondents (n=29). The survey had an average completion time of four minutes and 48 seconds.

Most respondents report working in healthcare for 11 or more years (70.6%; n=60) (Table 1).

**Table 1:** Years in healthcare physician associate survey respondents

Years in healthcare (n=85)	Actual N (%)
0-1 years	4 (4.7)
2-5 years	9 (10.6)
6-10 years	12 (14.1)
11+ years	60 (70.6)

The respondents mostly work in outpatient clinic settings (66.7%; n=52), with the remaining respondents practicing mainly in hospitals and emergency services. Primary care is the largest specialty represented (37%; n=27), with at least 20 additional specialties reported (Table 2).

**Table 2:** Practice characteristics of physician associate survey respondents

	Actual N (%)
Practice setting (n=78)	
Outpatient clinic	52 (66.7)
Hospital	18 (23)
Emergency services	8 (10.3)
Administrative/consulting (retired clinical)	2 (2.56)
Skilled nursing facility	1 (1.3)
Physical rehab inpatient	1 (1.3)
Inpatient rehab facility	1 (1.3)
Correctional facility	1 (1.3)
County detention center	1 (1.3)
Both outpatient and hospital	1 (1.3)
Primary clinical specialty (n=73)	
Primary care	27 (37)
Emergency department	10 (13.7)
Internal medicine	8 (11)
Orthopedics	7 (9.6)
Addiction medicine	5 (6.9)
Surgery	5 (6.9)
Pain management	3 (4.1)
Pediatrics	3 (4.1)
Urgent care	3 (4.1)
Physical medicine and rehabilitation (PM&R)	2 (2.6)
Obstetrics/gynecology	2 (2.8)
Urology	2 (2.6)
Dermatology	1 (1.28)
Pediatric neurosurgery	1 (1.28)
Interventional radiology	1 (1.28)
Education (non-clinical)	1 (1.28)
Pulmonary/critical care	1 (1.28)
Gastroenterology	1 (1.28)
Corrections medicine	1 (1.28)
Pulmonary sleep	1 (1.28)
Medical/surgical critical care	1 (1.28)
Neurology	1 (1.28)
ICU	1 (1.28)
Pediatric neurology	1 (1.28)
Hospitalist	1 (1.28)
Psychiatry	1 (1.28)
Rheumatology	1 (1.28)

Respondents represent 34 states. The three states with the highest representation are Michigan (16.7%; n=15), California (10%; n=9), and Pennsylvania (7.8%; n=7) (Table 3).

**Table 3:** Geographic distribution of physician associate survey respondents

State (n=90)	Actual N (%)
Michigan	15 (16.7)
California	9 (10)
Pennsylvania	7 (7.8)
Texas	5 (5.5)
Florida	5 (5.5)
Washington	3 (3.3)
Virginia	3 (3.3)
New York	3 (3.3)
West Virginia	3 (3.3)
Illinois	3 (3.3)
Wisconsin	2 (2.2)
Nevada	2 (2.2)
Colorado	2 (2.2)
Maryland	2 (2.2)
New Jersey	2 (2.2)
Georgia	2 (2.2)
Massachusetts	2 (2.2)
North Carolina	2 (2.2)
Alabama	1 (1.1)
Missouri	1 (1.1)
Minnesota	1 (1.1)
North Dakota	1 (1.1)
Tennessee	1 (1.1)
Ohio	1 (1.1)
Nebraska	1 (1.1)
Connecticut	1 (1.1)
Oregon	1 (1.1)
Maine	1 (1.1)
Arizona	1 (1.1)
Hawaii	1 (1.1)
Delaware	1 (1.1)
Idaho	1 (1.1)
Oklahoma	1 (1.1)
Arkansas	1 (1.1)

Clinical opioid prescribing

The majority of respondents (55.1%; n=49) report prescribing opioids for pain management only, while 19.1% (n=17) report prescribing opioids for pain and addiction treatment. A smaller group prescribe opioids only for addiction treatment (5.6%; n=5), and 20.2% (n=18) report not prescribing opioids at all.

The majority of PAs who completed the survey (61.8%; n=55) report that their opioid prescribing is usually short term/acute for periods of less than 30 days, while 18% (n=16) report they are usually prescribing opioids long term/chronic for more than 30 days. While the majority of respondents identify as prescribing opioids acutely, this may not account for the entirety of their opioid prescribing with some respondents engaged in both acute and chronic opioid prescribing.

Naloxone education, knowledge, and confidence

Most respondents affirm having received some form of formal education on naloxone (91.1%; n=82) and report at least some familiarity with national practice guidelines on naloxone prescribing (92.2%; n=83). Of those reporting some familiarity, 13.3% (n=12) consider themselves to be extremely knowledgeable on national practice guidelines for naloxone. Respondent confidence in knowledge of state-specific naloxone laws (e.g., third-party prescribing, standing orders) is split: 50.6% (n=43) report being confident, and 49.4%(n=42) report not being confident.

While the majority of all respondents (91.7%; n=77) indicate they would know how to administer naloxone in an emergency, a group of respondents (13.8%; n=11) indicate they did not feel confident in their understanding of how naloxone works.

Attitudes toward naloxone prescribing and normalization

All respondents to this question (100%; n=85) believe that naloxone is effective at reversing opioid overdose. Most respondents (96.5%; n=82) agree that patients at risk of overdose should always be offered naloxone. A strong majority support normalizing having naloxone on hand (95.3%; n=81) and believe that naloxone distribution increases community safety (94.1%; n=80). However, there are notable discrepancies in the responses regarding attitudes towards naloxone acceptance and prescribing. Despite the vast majority of respondents agreeing with the general statements regarding naloxone listed above, nearly three-quarters of respondents (75.3%; n=64) agree with the statement that prescribing naloxone might be "more worthwhile" for some patients than others, with 11.8% (n=10) reporting discomfort discussing naloxone with patients who use substances (e.g., marijuana, methamphetamine). A further 12.8% (n=11) of those PAs surveyed do not feel comfortable determining who should receive naloxone, and 12.5% (n=11) do not feel comfortable educating patients on naloxone.

The survey results suggest a great deal of contradictory attitudes even within individual respondents (Figure 1). This is illustrated by one respondent, a primary care PA. This PA has over 11 years of healthcare experience, reports working in a setting that supports naloxone education and distribution, and describes themselves as likely to prescribe naloxone to patients using substances. According to their responses, this respondent does not believe naloxone increases drug use or risky behavior and agrees patients at risk should be offered naloxone. At the same time, this PA also has concerns about how patients will perceive them when discussing naloxone for overdose and worries about potential misuse or abuse when prescribing naloxone. Negative responses to patient perception were not limited to a specific specialty or state.

**Figure 1:**
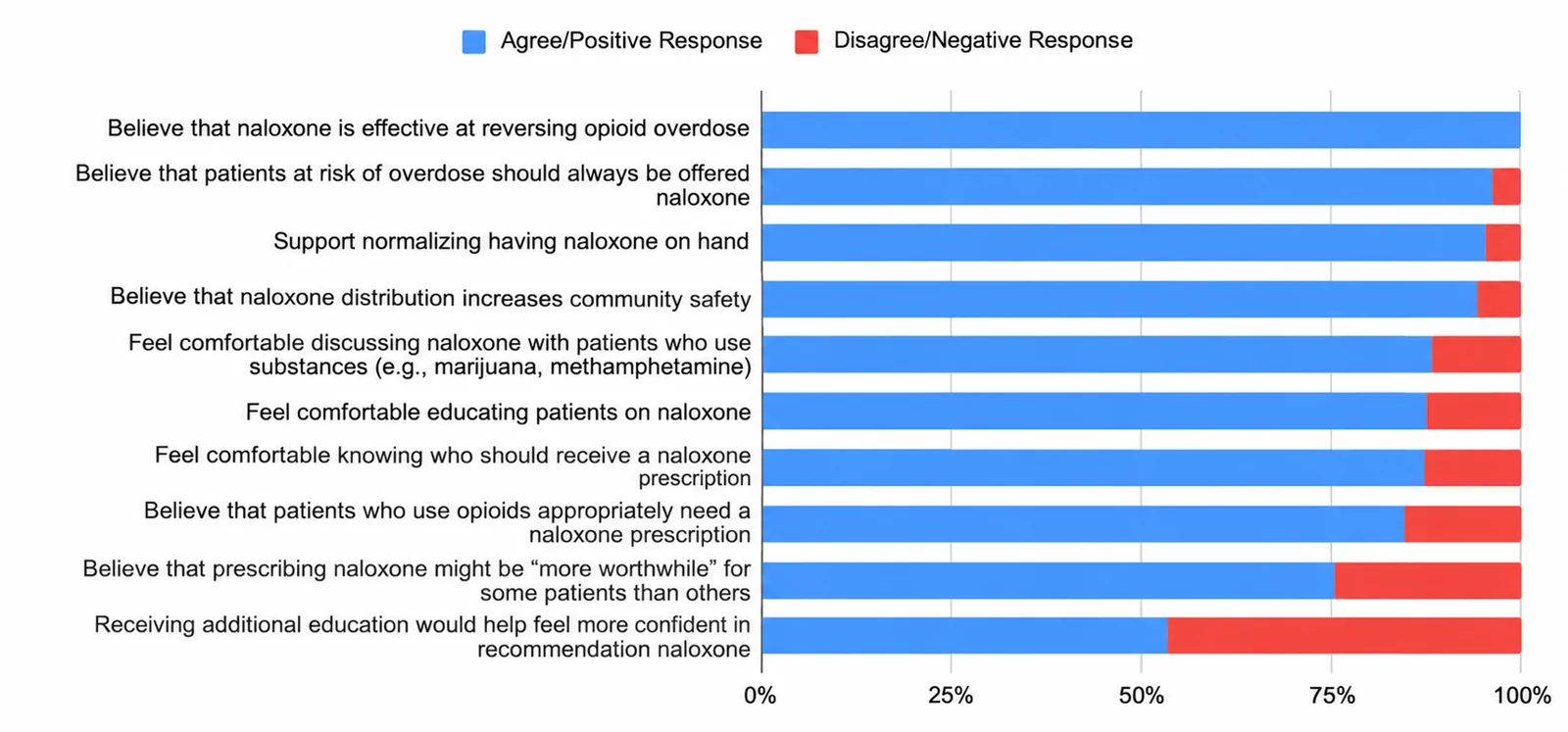
Juxtaposition of naloxone prescribing beliefs

Barriers to naloxone prescribing

Several barriers to naloxone prescribing were identified based on the survey responses. Time constraint was reported as a barrier with 51.1% (n=45) of respondents feeling that they do not have enough time during visits to discuss naloxone with patients. Concerns about patient perception are also a factor with 18.2% (n=16) of respondents who feel concerned about how patients will perceive them discussing naloxone for opioid overdose. When asked whether naloxone availability increases continued substance use or risk-taking, 4.9% (n=4) feel it increases such behaviors, 12.2% (n=10) feel it decreases them, 65.9% (n=54) report no perceived impact, and 17.1% (n=14) of respondents are unsure of its effect.

Legal concerns and workplace support

The majority of respondents report no major concerns about the legal implications of prescribing naloxone, with 100% (n=71) of respondents indicating they are neutral, disagree, or strongly disagree with the statement that they worry about such implications. Most respondents report institutional support for naloxone education and distribution in their workplaces (79.7%; n=55). It should be noted that one respondent (1.45%) reports that their workplace does not support naloxone distribution and education, the likely impact of which will extend beyond the individual respondent to include all providers associated with that organization. While not captured in the survey responses, this effect could also be expected in other workplaces or practice settings with similar policies discouraging the promotion of naloxone.

Educational insights

The majority of respondents (70%; n=61) indicated they would benefit from additional education about naloxone and overdose prevention with 19.8% (n=17) of those strongly agreeing they would benefit from additional education. Importantly, 53.5% (n=46) feel additional training would increase their confidence in recommending naloxone.

Qualitative themes from open-ended responses

Free-text comments revealed several themes including time constraints and patient education, stigma and patient perceptions, concerns about access and cost, normalization of naloxone in communities, clinical experience and observations, and patient behaviors and outcomes (Table 4).

**Table 4:** Themes and representative quotes on naloxone prescribing challenges

Theme	Representative quotes
Time constraints and patient education	"Limited time for patient education, especially when working against stigmas patients already hold about opiate use and naloxone. It takes much longer than medication education for a non-controlled substance."
	"Issue for me is seeing and prescribing it to enough patients to feel comfortable and more knowledgeable about the drug and its benefits. I've had a ton of 'education' that is not direct clinical cases."
Stigma and patient perceptions	"I do not want my patients to believe that I think they're addicts when I am only prescribing opiates for acute postoperative pain."
	"Patients misinterpreting my intentions (I'm assuming they're misusing their meds)."
	"Patient's not filling the prescription due to stigma or they are worried about future life insurance coverage."
	"No concerns about prescribing. Only concern about recommending is patients thinking I am making assumptions or judging, but I do my very best to let them know this is important for cases of accidental overdose."
Concerns about access and cost	"My only concern is when I prescribe it to patients with their narcotic medications, many will not pay for it or pick it up because of cost or because they don't think they can overdose."
	"Copays for commercial patients can be a concern."
Normalization and importance of naloxone	"It is a life-saving treatment that needs to be normalized."
	"I believe it should be mandatory stocked in all dorms and rental units around colleges!"
Clinical experience and observations	"I have administered Naloxone nasal and IM many times over the years and performed CPR due to respiratory distress from an overdose outside a facility while waiting on EMS. I wish Naloxone was packaged with AEDs!"
	"If someone is actually only using cannabis or cannabinoids, I am less concerned if they choose not to have naloxone than someone who is using opioids or other substances more likely to have fentanyl included."
Patient behavior and outcomes	"Patients overdosing again."
	"It normalizes opioid use/abuse."

Summary of results

The results provide insights into PA attitudes around naloxone prescribing, representing PAs from many states and specialties. PAs in this sample overwhelmingly recognize naloxone as an effective, life-saving intervention and generally support its normalization in the community. However, while some of the results are strongly favorable for naloxone prescribing, beliefs, and understanding, there is also the presence of conflicting viewpoints as well as identified barriers to increased adoption of naloxone practices. Important barriers including limited time, patient stigma, cost/insurance concerns, and gaps in familiarity with state laws were identified in survey answers. Many PA respondents indicated that additional education would increase their willingness and ability to prescribe naloxone to patients.

## Discussion

This survey highlights many important insights related to PA beliefs around naloxone prescribing. Although efforts to address the opioid epidemic have slowed the rise in overdose deaths beginning in 2021 to the present, there remains a high fatality rate that underscores the need for additional harm reduction strategies [2,3]. There remains a poor understanding of why US providers, particularly PAs, who prescribe opioids often fail to prescribe naloxone, despite its life-saving potential for those at risk of overdose. The gap between those patients on opioids and those without a naloxone prescription persists amid the ongoing opioid epidemic, despite the emphasized need for safer prescribing and harm reduction strategies. Naloxone prescribing rates have increased since 2018 but remain under-prescribed with one naloxone prescription dispensed for every 70 prescriptions of high-dose opioids [6,11,12].

The findings of this survey suggest that PAs across the country are largely familiar with national practice guidelines on naloxone prescribing. The responses also suggest that most, if not all, PAs believe that naloxone is effective at reversing opioid overdose and that patients at risk of overdose should always be offered naloxone. It also highlights that PAs support normalizing having naloxone on hand and believe that naloxone distribution increases community safety. Given these findings, despite generally favorable attitudes toward naloxone, PA respondents identified several perceived barriers that could interfere with naloxone prescribing.

The results of this survey align with previous research that has identified barriers such as time and cost that may influence naloxone prescribing with the majority of PAs feeling that they do not have enough time during visits to discuss naloxone [3,9]. Institutional support for naloxone education and distribution was not prominently identified as a barrier; however, some of the captured additional comments by several respondents give indication that organizational/workplace policies may act to significantly influence individual provider practices.

While current national practice guidelines support offering naloxone for all individuals prescribed opioids, this survey suggests that there is a belief among some PAs that patients who use opioids appropriately do not need naloxone and that prescribing naloxone might be more worthwhile for some patients than others [5]. The survey responses hint that this perception may be present among practicing PAs who report some discomfort discussing naloxone with patients who use substances, do not always feel comfortable knowing who should receive naloxone, and do not always feel comfortable educating patients on naloxone.

Stigma by healthcare providers, in this case PAs, may play a significant, though often unspoken, role in limiting naloxone access. Survey responses suggest that providers may fear offending patients by discussing naloxone or that it may signal distrust or may imply that a patient is at risk for misuse or overdose. These concerns may lead to hesitation in prescribing naloxone altogether, especially in primary care or pain management settings where long-term patient relationships are central. In turn, patients may exhibit stigma toward naloxone that is rooted in the perception that the provider suspects them of substance misuse or in instances where there is insufficient education about naloxone and opioid overdose by their provider [6]. There are also PA respondents who have concerns that naloxone availability could encourage continued or increase substance use.

Most of the surveyed PAs have received some form of formal education on naloxone. Yet strikingly, many agree that they would benefit from additional education and training on naloxone and overdose prevention and that such education would positively influence their confidence around naloxone prescribing. In addition to this identified opportunity is the need for additional education and availability of information on state-specific naloxone laws.

The findings from this exploratory study provide a foundation for future research examining state-level and practice-setting differences in opioid prescribing rates, as well as whether prescribing opioids for chronic versus acute conditions influences provider perceptions of naloxone. There is also a clear opportunity in the development of future clinical guidelines, education, and organizational policies targeting PAs that normalizes naloxone prescribing as a routine and evidence-based component of opioid therapy.

Limitations

As an exploratory survey-based study, this research has several limitations. Since distribution of the survey was conducted via social media platforms, overall response rate cannot accurately be determined. Respondents to this survey may not reflect the broader population of practicing PAs. There may be a selection bias in that those who responded to the survey are already more positive about the topic and the survey instrument may not capture the full range of factors that providers consider when prescribing naloxone. Although care was taken in framing the questions, there remains a possibility of unintended bias in wording or interpretation. The survey did not determine how many respondents are actually prescribing naloxone but instead focused on whether they prescribe opioids. It is also not clear what the exact distinction is between how many of the PA respondents may prescribe opioids both acutely and chronically. Additionally, while most respondents answered all survey questions, within the survey, participants could skip questions, and the generalizability of responses may be impacted if it is unclear why some questions were skipped. The survey was also available only in English.

## Conclusions

Opioid-related overdose mortality remains an important public health concern in the United States, and naloxone can save lives when it is available. When providers worry about enabling drug use or offending patients, they often hesitate to prescribe naloxone, which only adds to patient stigma. This study offers new insights on PA perceptions about naloxone prescribing. Most importantly, this new understanding highlights the need for increased education for providers and more time to educate patients on naloxone. The findings can be used to develop better training and policies that will reduce provider stigma and ensure more people at risk for opioid overdose have access to this life‑saving medication.

## Appendices

Survey

*Informed Consent Agreement* Please read this consent agreement carefully before you decide to participate in this study.

*Title of Investigation* Physician Associates Attitudes Regarding Prescribing of Naloxone for Treatment of Opioid Overdose

*Investigator* Amanda Novak MPAS, PAC Email: vhw48@su.edu

*Purpose of the Study* The purpose of this study is to examine the attitudes and beliefs that physician associates hold about prescribing naloxone for the treatment of opioid overdose. You must be at least 18 years of age and have graduated from an accredited PA program.  

*Procedures* If you agree to participate, you will complete an online survey in which you will answer some questions about your current practices and prescribing of naloxone products. This will take no longer than 10 minutes to complete. All responses will be anonymous unless you opt in to providing contact information in the form of an email address.  

*Discomforts and Risks* Participation in this survey is voluntary. The way that you answer these questions will not impact the ability to care for patients or your reputation. All the responses that you provide will be kept confidential; your name and other identifying information will not be collected or stored.

*Potential Benefits* There are no direct benefits to you for participating, but your responses may help improve the understanding of naloxone prescribing. 

*Period of Time Required* This is a 33-question survey, which should take approximately 7-10 minutes to complete.

If you have questions about this study, please contact: Principal investigator: Amanda Novak, at hw48@su.edu Faculty advisors: Michelle Gruver at msalerno@su.edu or Kirsten Brondstater at kbarker@su.edu

You may report a concern about a study, ask questions about a study, ask questions about your rights as a research subject, or report a research-related injury by contacting the Institutional Review Board listed below. IRB Compliance Coordinator, sucomply@su.edu or IRB Chair, irbchair@su.edu 

You may print a copy of this page for your records.

By clicking OK, you acknowledge that you are over 18 years of age and are agreeing to participate in the research study described above. Because your name and other identifying information are not being collected, you cannot withdraw your participation after you have submitted your survey. 

What is your current role?

Multiple choice Physician Physician Associate Nurse Practitioner Pharmacist Other (please specify): __________

How many years have you worked in healthcare?

Multiple choice 0-1 years 2-5 years 6-10 years 11+ years I am a student

In what setting do you primarily work?

Multiple choice Hospital Outpatient Clinic Emergency Services Community Pharmacy Other (please specify): __________

What is your primary clinical specialty?

Multiple choice Family Medicine Internal Medicine Emergency Medicine Pediatrics Pain Management Surgery PM&R Orthopedics Psychiatry Obstetrics/Gynecology Dermatology Addiction Medicine Other (please specify): __________

What state do you work in? (primary location)

Short answer

Do you prescribe opioids for the treatment of pain or addiction in your practice?

Multiple choice Pain Addiction Neither Both

If you prescribe opioids, are your prescriptions usually:

Multiple choice Acute (less than 30 days) Chronic (more than 30 days) Not applicable

Have you received formal education on opioid overdose prevention or naloxone use? Multiple choice Yes No Unsure

Are you familiar with national practice guidelines regarding the prescribing of naloxone?

Multiple choice Extremely knowledgeable Moderately knowledgeable Slightly knowledgeable Not at all knowledgeable Unsure

Please indicate your response (Yes/No):

Do you feel confident in your knowledge about how naloxone works? Do you feel you know how to administer naloxone in an emergency? Do you feel confident in your understanding of your state's laws regarding naloxone (i.e., third-party prescribing, standing orders, etc.)? Do you believe naloxone is effective at reversing opioid overdoses? Do you feel patients at risk of overdose should always be offered a prescription for naloxone? Do you feel naloxone distribution increases community safety? Do you feel comfortable discussing naloxone with patients who use substances such as marijuana, methamphetamine, etc.? Do you feel prescribing naloxone to some patients is more worthwhile than others? Do you feel patients who use their opioids appropriately need a naloxone prescription? Do you feel comfortable knowing who should receive a naloxone prescription? Do you feel comfortable educating your patients on naloxone? Do you feel concerned about how your patients will perceive you discussing naloxone for opioid overdose with them? Do you feel you have enough time during appointments to discuss naloxone? Do you have access to naloxone in your workplace? Do you feel having naloxone on hand should be normalized, like an EpiPen or an inhaler, in case of emergency?

What impact does naloxone have on continued substance use and/or substance-related risk-taking?

Multiple choice Increases continued drug use or risky behavior Decreases continued drug use or risky behavior Has no change in continued drug use or risky behavior Unsure

If a patient has a history of misusing opioids or other substances (such as marijuana, methamphetamine, etc.), how likely are you to prescribe naloxone?

Multiple choice Very likely Likely Neither likely nor unlikely Unlikely Very unlikely

My workplace supports naloxone education and distribution.

Multiple choice Strongly agree Agree Neither agree nor disagree Disagree Strongly disagree

I worry about legal or professional consequences of prescribing naloxone.

Multiple choice Strongly agree Agree Neither agree nor disagree Disagree Strongly disagree

I would benefit from additional education about naloxone and overdose prevention.

Multiple choice Strongly agree Agree Neither agree nor disagree Disagree Strongly disagree

Would receiving additional education help you feel more confident in recommending naloxone?

Multiple choice Yes No - I already feel comfortable. No - nothing would make me feel more comfortable prescribing naloxone. 

What are your biggest concerns about prescribing or recommending naloxone?

Paragraph response

Would you be open to being contacted for additional information?

Multiple choice Yes (If yes, please leave your email below) No If yes, please provide your email address (optional):

Short answer

Do you have any additional comments? (optional)

Short answer
